# 2,6-Bis[1-(2-methyl­phenyl­imino)eth­yl]pyridine

**DOI:** 10.1107/S1600536809020522

**Published:** 2009-06-06

**Authors:** Rui-Qing Fan, Xiao-Dong Ding, Guang-Peng Zhou, Yu-Lin Yang

**Affiliations:** a Department of Chemistry, Harbin Institute of Technology, Harbin 150001, People’s Republic of China

## Abstract

The mol­ecule of the title compound, C_23_H_23_N_3_, which was synthesized by the condensation reaction between 2,6-diacetyl­pyridine and 2-dimethyl­aniline, adopts an *E* configuration about both C=N imine bonds. The dihedral angles formed by the benzene rings with the pyridine ring are 89.68 (5) and 53.62 (6)°.

## Related literature

For the applications of pyridine-based ligands in sensor technologies and electro-luminescent devices, see: Tang & Vanslyke (1987 ▶); Wang (2001 ▶). For the crystal structures of related compounds, see: Mentes *et al.* (2001 ▶); Huang *et al.* (2006 ▶). For the synthesis, see: Fan *et al.* (2004 ▶).
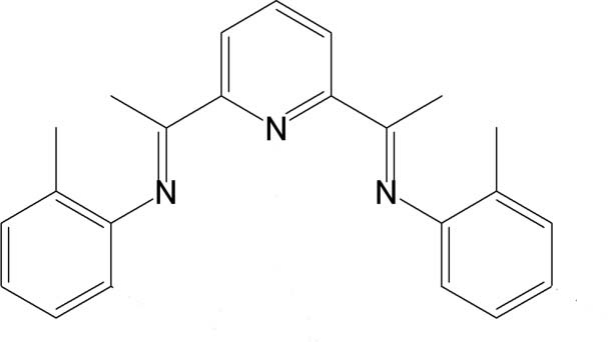


## Experimental

### 

#### Crystal data

C_23_H_23_N_3_
*M* *_r_* = 341.44
Monoclinic, 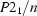

*a* = 12.966 (3) Å
*b* = 11.304 (2) Å
*c* = 14.767 (3) Å
β = 115.62 (3)°
*V* = 1951.6 (8) Å^3^
*Z* = 4
Mo *K*α radiation
μ = 0.07 mm^−1^
*T* = 193 K
0.56 × 0.41 × 0.36 mm

#### Data collection

Bruker SMART APEX CCD area-detector diffractometer
Absorption correction: multi-scan (*SADABS*; Bruker, 2000 ▶) *T*_min_ = 0.960, *T*_max_ = 0.972
18526 measured reflections
4435 independent reflections
2804 reflections with *I* > 2σ(*I*)
*R*_int_ = 0.037

#### Refinement

*R*[*F* ^2^ > 2σ(*F* ^2^)] = 0.051
*wR*(*F* ^2^) = 0.157
*S* = 1.06
4435 reflections
235 parameters
H-atom parameters constrained
Δρ_max_ = 0.22 e Å^−3^
Δρ_min_ = −0.16 e Å^−3^

### 

Data collection: *SMART* (Bruker, 2000 ▶); cell refinement: *SAINT* (Bruker, 2000 ▶); data reduction: *SAINT*; program(s) used to solve structure: *SHELXS97* (Sheldrick, 2008 ▶); program(s) used to refine structure: *SHELXL97* (Sheldrick, 2008 ▶); molecular graphics: *SHELXTL* (Sheldrick, 2008 ▶); software used to prepare material for publication: *SHELXTL*.

## Supplementary Material

Crystal structure: contains datablocks I. DOI: 10.1107/S1600536809020522/rz2329sup1.cif

Structure factors: contains datablocks I. DOI: 10.1107/S1600536809020522/rz2329Isup2.hkl

Additional supplementary materials:  crystallographic information; 3D view; checkCIF report

## Figures and Tables

**Fig. 1.**
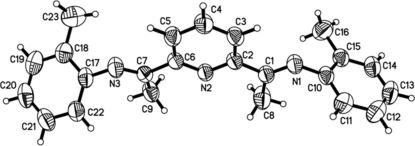
The molecular structire of the title compound showing the atom-labelling scheme. Displacement ellipsoids are drawn at the 50% probability level.

**Fig. 2.**
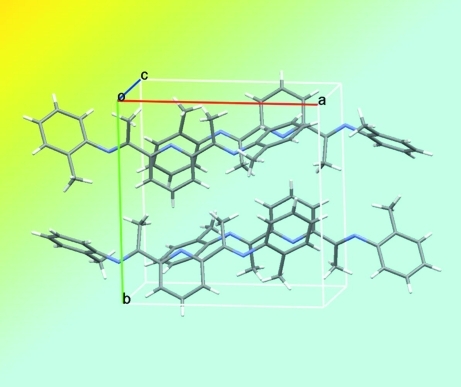
Packing diagram of the title compound viewed along the c axis.

## supplementary crystallographic information

### Comment

Luminescent coordination compounds based on pyridine-type ligands have attracted intensive attention due to their potential application in areas of sensor technologies and electro-luminescent devices (Tang & Vanslyke, 1987; Wang, 2001). In order to explore potential luminescent complexes of this type, we prepared a series of bis(iminoalkyl)pyridine ligands by the condensation reaction of 2,6-diacetylpyridine with the corresponding aniline in methanol (Fan *et al.*, 2004). It is still challenging to design and rationally synthesize ligands with unique structures and functions. In this regard, we report herein the synthesis and crystal structure of the title compound.

The molecule of the title compound (Fig. 1) possesses an approximate *C*_s_ symmetry about a plane bisecting the pyridine ring. The pyridine ring is coplanar with the two imino groups, which show typical C═N double bond character (1.2606 (18) and 1.2674 (19) Å for N1═C1 and N3═C7, respectively). These values are in good agreement with those observed in 2,6-bis[1-(phenylimino)ethyl]pyridine (1.266 (4) Å; Mentes *et al.*, 2001) and in 2,6-bis[1-(2,6-dimethylphenylimino)ethyl]pyridine (1.265 (2) and 1.271 (2) Å; Huang *et al.*, 2006). The dihedral angles between the C10–C15 and C17–C22 benzene rings and the pyridine ring are 89.68 (5) and 53.62 (6)°, respectively. The crystal packing (Fig. 2) is stabilized only by van der Waals interactions.

### Experimental

The title compound was synthesized according to the literatute method (Fan *et al.*, 2004). To a solution of 2,6-diacetylpyridine (1.1 g, 6.7 mmol) in absolute methanol (25 ml) was added 2-dimethylaniline (2.2 ml, 20.5 mmol). After the addition of several drops of formic acid, the reaction mixture was refluxed for 24 h and then allowed to cool down to room temperature. The crude product precipitated as yellow powder. Yellow block crystals suitable for X-ray diffraction analysis were obtained by slow evaporation of a methanol solution in 85% yield (1.96 g).

### Refinement

All H atoms were positioned geometrically with C—H = 0.93–0.96 Å, and allowed to ride on their parent atoms with *U*_iso_(H) = 1.2 *U*_eq_(C) or 1.5 *U*_eq_(C) for methyl H atoms.

### Crystal data

**Table:**

C_23_H_23_N_3_	*F*(000) = 728
*M**_r_* = 341.44	*D*_x_ = 1.162 Mg m^−^^3^
Monoclinic, *P*2_1_/*n*	Mo *K*α radiation, λ = 0.71073 Å
Hall symbol: -P 2yn	Cell parameters from 18526 reflections
*a* = 12.966 (3) Å	θ = 3.1–27.5°
*b* = 11.304 (2) Å	µ = 0.07 mm^−^^1^
*c* = 14.767 (3) Å	*T* = 193 K
β = 115.62 (3)°	Block, yellow
*V* = 1951.6 (8) Å^3^	0.56 × 0.41 × 0.36 mm
*Z* = 4	

### Data collection

**Table:**

Bruker SMART APEX CCD area-detector diffractometer	4435 independent reflections
Radiation source: fine-focus sealed tube	2804 reflections with *I* > 2σ(*I*)
graphite	*R*_int_ = 0.037
phi and ω scans	θ_max_ = 27.5°, θ_min_ = 3.1°
Absorption correction: multi-scan (*SADABS*; Bruker, 2000)	*h* = −16→16
*T*_min_ = 0.960, *T*_max_ = 0.972	*k* = −14→13
18526 measured reflections	*l* = −19→19

### Refinement

**Table:**

Refinement on *F*^2^	Primary atom site location: structure-invariant direct methods
Least-squares matrix: full	Secondary atom site location: difference Fourier map
*R*[*F*^2^ > 2σ(*F*^2^)] = 0.051	Hydrogen site location: inferred from neighbouring sites
*wR*(*F*^2^) = 0.157	H-atom parameters constrained
*S* = 1.06	*w* = 1/[σ^2^(*F*_o_^2^) + (0.0848*P*)^2^ + 0.041*P*] where *P* = (*F*_o_^2^ + 2*F*_c_^2^)/3
4435 reflections	(Δ/σ)_max_ < 0.001
235 parameters	Δρ_max_ = 0.22 e Å^−^^3^
0 restraints	Δρ_min_ = −0.16 e Å^−^^3^

### Special details

**Table:**

Geometry. All e.s.d.'s (except the e.s.d. in the dihedral angle between two l.s. planes) are estimated using the full covariance matrix. The cell e.s.d.'s are taken into account individually in the estimation of e.s.d.'s in distances, angles and torsion angles; correlations between e.s.d.'s in cell parameters are only used when they are defined by crystal symmetry. An approximate (isotropic) treatment of cell e.s.d.'s is used for estimating e.s.d.'s involving l.s. planes.
Refinement. Refinement of *F*^2^ against ALL reflections. The weighted *R*-factor *wR* and goodness of fit *S* are based on *F*^2^, conventional *R*-factors *R* are based on *F*, with *F* set to zero for negative *F*^2^. The threshold expression of *F*^2^ > σ(*F*^2^) is used only for calculating *R*-factors(gt) *etc*. and is not relevant to the choice of reflections for refinement. *R*-factors based on *F*^2^ are statistically about twice as large as those based on *F*, and *R*- factors based on ALL data will be even larger.

### Fractional atomic coordinates and isotropic or equivalent isotropic displacement parameters (Å^2^)

**Table:**

	*x*	*y*	*z*	*U*_iso_*/*U*_eq_	
N2	0.27847 (10)	0.74889 (10)	0.16139 (9)	0.0458 (3)	
N1	−0.01920 (11)	0.79913 (11)	0.03667 (10)	0.0527 (3)	
C6	0.37804 (12)	0.80381 (12)	0.18433 (11)	0.0448 (4)	
N3	0.57761 (11)	0.77842 (11)	0.25205 (11)	0.0565 (4)	
C2	0.18145 (13)	0.80916 (12)	0.10943 (11)	0.0457 (4)	
C1	0.07290 (13)	0.74430 (12)	0.08606 (11)	0.0476 (4)	
C7	0.48407 (13)	0.73369 (12)	0.24317 (11)	0.0466 (4)	
C3	0.18154 (13)	0.92576 (13)	0.08007 (12)	0.0524 (4)	
H3B	0.1130	0.9656	0.0444	0.063*	
C5	0.38449 (14)	0.92051 (13)	0.15758 (13)	0.0543 (4)	
H5A	0.4550	0.9567	0.1751	0.065*	
C10	−0.12858 (13)	0.74706 (12)	0.00704 (12)	0.0489 (4)	
C17	0.68397 (13)	0.72253 (14)	0.30780 (13)	0.0523 (4)	
C15	−0.18677 (13)	0.75912 (12)	0.06699 (12)	0.0522 (4)	
C4	0.28403 (14)	0.98142 (13)	0.10444 (13)	0.0586 (4)	
H4A	0.2858	1.0595	0.0853	0.070*	
C14	−0.29933 (15)	0.72146 (15)	0.02802 (15)	0.0620 (5)	
H14A	−0.3395	0.7298	0.0668	0.074*	
C22	0.71360 (14)	0.61751 (15)	0.27656 (14)	0.0633 (5)	
H22A	0.6602	0.5788	0.2202	0.076*	
C9	0.47073 (15)	0.62046 (14)	0.28932 (15)	0.0633 (5)	
H9A	0.5443	0.5840	0.3248	0.095*	
H9B	0.4213	0.5681	0.2375	0.095*	
H9C	0.4381	0.6366	0.3353	0.095*	
C18	0.76376 (15)	0.78082 (15)	0.39198 (13)	0.0579 (4)	
C8	0.08130 (16)	0.61968 (15)	0.12366 (17)	0.0764 (6)	
H8A	0.0058	0.5882	0.1037	0.115*	
H8B	0.1217	0.6191	0.1956	0.115*	
H8C	0.1217	0.5721	0.0958	0.115*	
C13	−0.35352 (16)	0.67238 (16)	−0.06562 (16)	0.0697 (5)	
H13A	−0.4295	0.6487	−0.0902	0.084*	
C11	−0.18236 (16)	0.69527 (15)	−0.08650 (14)	0.0634 (5)	
H11A	−0.1426	0.6850	−0.1254	0.076*	
C20	0.90039 (16)	0.62620 (19)	0.41061 (16)	0.0742 (6)	
H20A	0.9733	0.5946	0.4454	0.089*	
C12	−0.29465 (17)	0.65852 (17)	−0.12285 (15)	0.0721 (5)	
H12A	−0.3304	0.6243	−0.1862	0.087*	
C19	0.87169 (16)	0.72988 (18)	0.44214 (14)	0.0708 (5)	
H19A	0.9259	0.7674	0.4988	0.085*	
C16	−0.12855 (18)	0.81252 (18)	0.17075 (15)	0.0768 (6)	
H16A	−0.1809	0.8138	0.2009	0.115*	
H16B	−0.1049	0.8918	0.1659	0.115*	
H16C	−0.0629	0.7659	0.2114	0.115*	
C21	0.82176 (17)	0.56955 (18)	0.32815 (17)	0.0744 (5)	
H21A	0.8407	0.4990	0.3066	0.089*	
C23	0.7334 (2)	0.89394 (18)	0.42839 (17)	0.0874 (6)	
H23A	0.6561	0.9154	0.3847	0.131*	
H23B	0.7842	0.9558	0.4283	0.131*	
H23C	0.7407	0.8829	0.4953	0.131*	

### Atomic displacement parameters (Å^2^)

**Table:**

	*U*^11^	*U*^22^	*U*^33^	*U*^12^	*U*^13^	*U*^23^
N2	0.0389 (7)	0.0437 (7)	0.0531 (7)	0.0021 (5)	0.0183 (6)	0.0030 (5)
N1	0.0407 (7)	0.0476 (7)	0.0659 (9)	0.0017 (5)	0.0193 (6)	0.0079 (6)
C6	0.0412 (8)	0.0442 (8)	0.0508 (8)	0.0034 (6)	0.0217 (7)	0.0030 (6)
N3	0.0409 (8)	0.0550 (7)	0.0719 (9)	0.0030 (6)	0.0227 (7)	0.0109 (6)
C2	0.0413 (8)	0.0460 (8)	0.0493 (8)	0.0043 (6)	0.0191 (7)	0.0026 (6)
C1	0.0423 (8)	0.0458 (8)	0.0533 (9)	0.0019 (6)	0.0193 (7)	0.0031 (6)
C7	0.0421 (8)	0.0441 (7)	0.0537 (9)	0.0027 (6)	0.0208 (7)	−0.0005 (6)
C3	0.0439 (9)	0.0460 (8)	0.0631 (10)	0.0072 (6)	0.0191 (7)	0.0075 (7)
C5	0.0434 (9)	0.0472 (8)	0.0721 (11)	−0.0002 (6)	0.0247 (8)	0.0068 (7)
C10	0.0394 (8)	0.0399 (7)	0.0619 (9)	0.0040 (6)	0.0166 (7)	0.0108 (6)
C17	0.0384 (8)	0.0541 (9)	0.0654 (10)	0.0012 (7)	0.0234 (8)	0.0113 (7)
C15	0.0446 (9)	0.0435 (8)	0.0658 (10)	0.0024 (6)	0.0212 (8)	0.0074 (7)
C4	0.0521 (10)	0.0421 (8)	0.0790 (11)	0.0036 (7)	0.0260 (8)	0.0122 (7)
C14	0.0454 (9)	0.0622 (10)	0.0774 (12)	0.0018 (8)	0.0256 (9)	0.0112 (9)
C22	0.0455 (9)	0.0639 (10)	0.0785 (12)	0.0029 (8)	0.0248 (9)	0.0004 (8)
C9	0.0487 (10)	0.0548 (9)	0.0876 (13)	0.0091 (7)	0.0305 (9)	0.0182 (8)
C18	0.0542 (10)	0.0614 (9)	0.0583 (10)	−0.0042 (8)	0.0245 (8)	0.0091 (7)
C8	0.0511 (10)	0.0542 (10)	0.1118 (16)	0.0021 (8)	0.0239 (10)	0.0249 (10)
C13	0.0429 (9)	0.0704 (11)	0.0806 (13)	−0.0085 (8)	0.0125 (9)	0.0122 (10)
C11	0.0589 (11)	0.0667 (10)	0.0621 (11)	−0.0051 (8)	0.0238 (9)	0.0013 (8)
C20	0.0415 (10)	0.0890 (14)	0.0863 (14)	0.0123 (9)	0.0222 (10)	0.0223 (11)
C12	0.0647 (12)	0.0717 (11)	0.0630 (11)	−0.0148 (9)	0.0116 (10)	−0.0010 (9)
C19	0.0518 (11)	0.0886 (13)	0.0613 (11)	−0.0094 (9)	0.0143 (9)	0.0121 (9)
C16	0.0730 (13)	0.0819 (13)	0.0805 (14)	−0.0175 (10)	0.0380 (11)	−0.0189 (10)
C21	0.0567 (11)	0.0729 (11)	0.1024 (15)	0.0155 (9)	0.0428 (11)	0.0108 (11)
C23	0.1025 (18)	0.0732 (12)	0.0791 (14)	−0.0003 (12)	0.0323 (13)	−0.0034 (10)

### Geometric parameters (Å, °)

**Table:**

N2—C6	1.3374 (18)	C22—H22A	0.9300
N2—C2	1.3420 (18)	C9—H9A	0.9600
N1—C1	1.2606 (18)	C9—H9B	0.9600
N1—C10	1.4187 (19)	C9—H9C	0.9600
C6—C5	1.390 (2)	C18—C19	1.394 (3)
C6—C7	1.497 (2)	C18—C23	1.504 (3)
N3—C7	1.2674 (19)	C8—H8A	0.9600
N3—C17	1.413 (2)	C8—H8B	0.9600
C2—C3	1.388 (2)	C8—H8C	0.9600
C2—C1	1.489 (2)	C13—C12	1.371 (3)
C1—C8	1.501 (2)	C13—H13A	0.9300
C7—C9	1.495 (2)	C11—C12	1.380 (3)
C3—C4	1.370 (2)	C11—H11A	0.9300
C3—H3B	0.9300	C20—C21	1.363 (3)
C5—C4	1.379 (2)	C20—C19	1.371 (3)
C5—H5A	0.9300	C20—H20A	0.9300
C10—C11	1.380 (2)	C12—H12A	0.9300
C10—C15	1.396 (2)	C19—H19A	0.9300
C17—C22	1.387 (2)	C16—H16A	0.9600
C17—C18	1.392 (2)	C16—H16B	0.9600
C15—C14	1.384 (2)	C16—H16C	0.9600
C15—C16	1.511 (3)	C21—H21A	0.9300
C4—H4A	0.9300	C23—H23A	0.9600
C14—C13	1.369 (3)	C23—H23B	0.9600
C14—H14A	0.9300	C23—H23C	0.9600
C22—C21	1.385 (2)		
			
C6—N2—C2	118.22 (12)	C7—C9—H9C	109.5
C1—N1—C10	123.03 (12)	H9A—C9—H9C	109.5
N2—C6—C5	122.59 (13)	H9B—C9—H9C	109.5
N2—C6—C7	116.43 (12)	C17—C18—C19	117.84 (17)
C5—C6—C7	120.96 (13)	C17—C18—C23	120.89 (17)
C7—N3—C17	122.08 (13)	C19—C18—C23	121.26 (18)
N2—C2—C3	122.25 (14)	C1—C8—H8A	109.5
N2—C2—C1	116.17 (12)	C1—C8—H8B	109.5
C3—C2—C1	121.58 (13)	H8A—C8—H8B	109.5
N1—C1—C2	117.13 (13)	C1—C8—H8C	109.5
N1—C1—C8	125.07 (14)	H8A—C8—H8C	109.5
C2—C1—C8	117.79 (13)	H8B—C8—H8C	109.5
N3—C7—C9	126.06 (14)	C14—C13—C12	119.32 (17)
N3—C7—C6	116.53 (13)	C14—C13—H13A	120.3
C9—C7—C6	117.37 (13)	C12—C13—H13A	120.3
C4—C3—C2	119.07 (14)	C10—C11—C12	120.57 (19)
C4—C3—H3B	120.5	C10—C11—H11A	119.7
C2—C3—H3B	120.5	C12—C11—H11A	119.7
C4—C5—C6	118.51 (14)	C21—C20—C19	119.81 (17)
C4—C5—H5A	120.7	C21—C20—H20A	120.1
C6—C5—H5A	120.7	C19—C20—H20A	120.1
C11—C10—C15	119.90 (15)	C13—C12—C11	120.05 (18)
C11—C10—N1	119.33 (16)	C13—C12—H12A	120.0
C15—C10—N1	120.41 (15)	C11—C12—H12A	120.0
C22—C17—C18	119.76 (15)	C20—C19—C18	122.03 (18)
C22—C17—N3	121.99 (15)	C20—C19—H19A	119.0
C18—C17—N3	118.04 (15)	C18—C19—H19A	119.0
C14—C15—C10	117.91 (16)	C15—C16—H16A	109.5
C14—C15—C16	121.28 (17)	C15—C16—H16B	109.5
C10—C15—C16	120.81 (15)	H16A—C16—H16B	109.5
C3—C4—C5	119.35 (14)	C15—C16—H16C	109.5
C3—C4—H4A	120.3	H16A—C16—H16C	109.5
C5—C4—H4A	120.3	H16B—C16—H16C	109.5
C13—C14—C15	122.21 (18)	C20—C21—C22	119.73 (18)
C13—C14—H14A	118.9	C20—C21—H21A	120.1
C15—C14—H14A	118.9	C22—C21—H21A	120.1
C21—C22—C17	120.83 (17)	C18—C23—H23A	109.5
C21—C22—H22A	119.6	C18—C23—H23B	109.5
C17—C22—H22A	119.6	H23A—C23—H23B	109.5
C7—C9—H9A	109.5	C18—C23—H23C	109.5
C7—C9—H9B	109.5	H23A—C23—H23C	109.5
H9A—C9—H9B	109.5	H23B—C23—H23C	109.5
			
C2—N2—C6—C5	0.8 (2)	C11—C10—C15—C14	−2.1 (2)
C2—N2—C6—C7	179.43 (13)	N1—C10—C15—C14	170.93 (13)
C6—N2—C2—C3	−0.6 (2)	C11—C10—C15—C16	178.34 (15)
C6—N2—C2—C1	−179.79 (13)	N1—C10—C15—C16	−8.6 (2)
C10—N1—C1—C2	178.32 (14)	C2—C3—C4—C5	−0.1 (3)
C10—N1—C1—C8	−2.3 (3)	C6—C5—C4—C3	0.3 (3)
N2—C2—C1—N1	−179.50 (14)	C10—C15—C14—C13	0.8 (2)
C3—C2—C1—N1	1.3 (2)	C16—C15—C14—C13	−179.72 (16)
N2—C2—C1—C8	1.0 (2)	C18—C17—C22—C21	0.1 (3)
C3—C2—C1—C8	−178.12 (16)	N3—C17—C22—C21	174.74 (16)
C17—N3—C7—C9	1.0 (3)	C22—C17—C18—C19	−0.2 (2)
C17—N3—C7—C6	178.40 (14)	N3—C17—C18—C19	−175.06 (15)
N2—C6—C7—N3	169.72 (14)	C22—C17—C18—C23	−179.14 (17)
C5—C6—C7—N3	−11.6 (2)	N3—C17—C18—C23	6.0 (2)
N2—C6—C7—C9	−12.7 (2)	C15—C14—C13—C12	0.7 (3)
C5—C6—C7—C9	165.96 (16)	C15—C10—C11—C12	2.1 (2)
N2—C2—C3—C4	0.3 (2)	N1—C10—C11—C12	−171.07 (15)
C1—C2—C3—C4	179.40 (15)	C14—C13—C12—C11	−0.8 (3)
N2—C6—C5—C4	−0.6 (2)	C10—C11—C12—C13	−0.5 (3)
C7—C6—C5—C4	−179.19 (15)	C21—C20—C19—C18	−0.4 (3)
C1—N1—C10—C11	−93.60 (19)	C17—C18—C19—C20	0.4 (3)
C1—N1—C10—C15	93.31 (19)	C23—C18—C19—C20	179.31 (19)
C7—N3—C17—C22	68.2 (2)	C19—C20—C21—C22	0.3 (3)
C7—N3—C17—C18	−117.05 (18)	C17—C22—C21—C20	−0.1 (3)
